# MetaVelvet-DL: a MetaVelvet deep learning extension for de novo metagenome assembly

**DOI:** 10.1186/s12859-020-03737-6

**Published:** 2021-06-02

**Authors:** Kuo-ching Liang, Yasubumi Sakakibara

**Affiliations:** Department of Biosciences and Informatics, Keio University, 3-14-1 Hiyoshi, Kohoku-ku, Yokohama, 223-8522 Japan

**Keywords:** Metagenome analysis, de novo assembly, Deep learning, de Bruijn graph, Long short-term memory, Convolutional neural network

## Abstract

**Background:**

The increasing use of whole metagenome sequencing has spurred the need to improve de novo assemblers to facilitate the discovery of unknown species and the analysis of their genomic functions. MetaVelvet-SL is a short-read de novo metagenome assembler that partitions a multi-species de Bruijn graph into single-species sub-graphs. This study aimed to improve the performance of MetaVelvet-SL by using a deep learning-based model to predict the partition nodes in a multi-species de Bruijn graph.

**Results:**

This study showed that the recent advances in deep learning offer the opportunity to better exploit sequence information and differentiate genomes of different species in a metagenomic sample. We developed an extension to MetaVelvet-SL, which we named MetaVelvet-DL, that builds an end-to-end architecture using Convolutional Neural Network and Long Short-Term Memory units. The deep learning model in MetaVelvet-DL can more accurately predict how to partition a de Bruijn graph than the Support Vector Machine-based model in MetaVelvet-SL can. Assembly of the Critical Assessment of Metagenome Interpretation (CAMI) dataset showed that after removing chimeric assemblies, MetaVelvet-DL produced longer single-species contigs, with less misassembled contigs than MetaVelvet-SL did.

**Conclusions:**

MetaVelvet-DL provides more accurate de novo assemblies of whole metagenome data. The authors believe that this improvement can help in furthering the understanding of microbiomes by providing a more accurate description of the metagenomic samples under analysis.

## Background

**Fig. 1:**
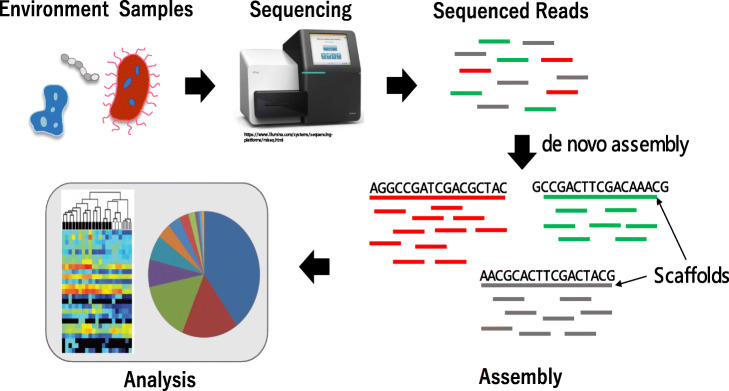
Workflow for de novo metagenome assembly

Recent advances in metagenome sequencing technologies and computational tools have allowed us to begin understanding how microbial communities can affect and be affected by their environment. With the improvement of high-throughput sequencing technologies, whole genome sequencing (WGS) has become an important tool for metagenomics analysis. It has several advantages over traditional 16S rRNA analysis, including more reliable species identification and gene prediction [1]. WGS data can be assembled with the help of reference genomes when the species are well presented in genome databases. However, metagenomic samples typically have large numbers of species of unknown identity and thus, the reference genome-based approach may fail to discover novel species or important variations within a species. De novo assembly, which does not require a reference genome, is useful in such cases. Figure 1 shows a typical workflow of de novo metagenome assembly. The metagenomics sample, containing large numbers of various bacterial species, is sequenced. Then the sequence reads are assembled into contigs in a de novo manner, typically using some graph-based approaches. Finally, the assembled genome is used for downstream analysis, such as binning and functional analysis. However, the assembly of multiple genomes from mixed sequence reads is challenging because the number of genomes and the coverage of each genome are initially unknown, and the coverage distribution is nonhomogeneous and potentially skewed.

**Fig. 2:**
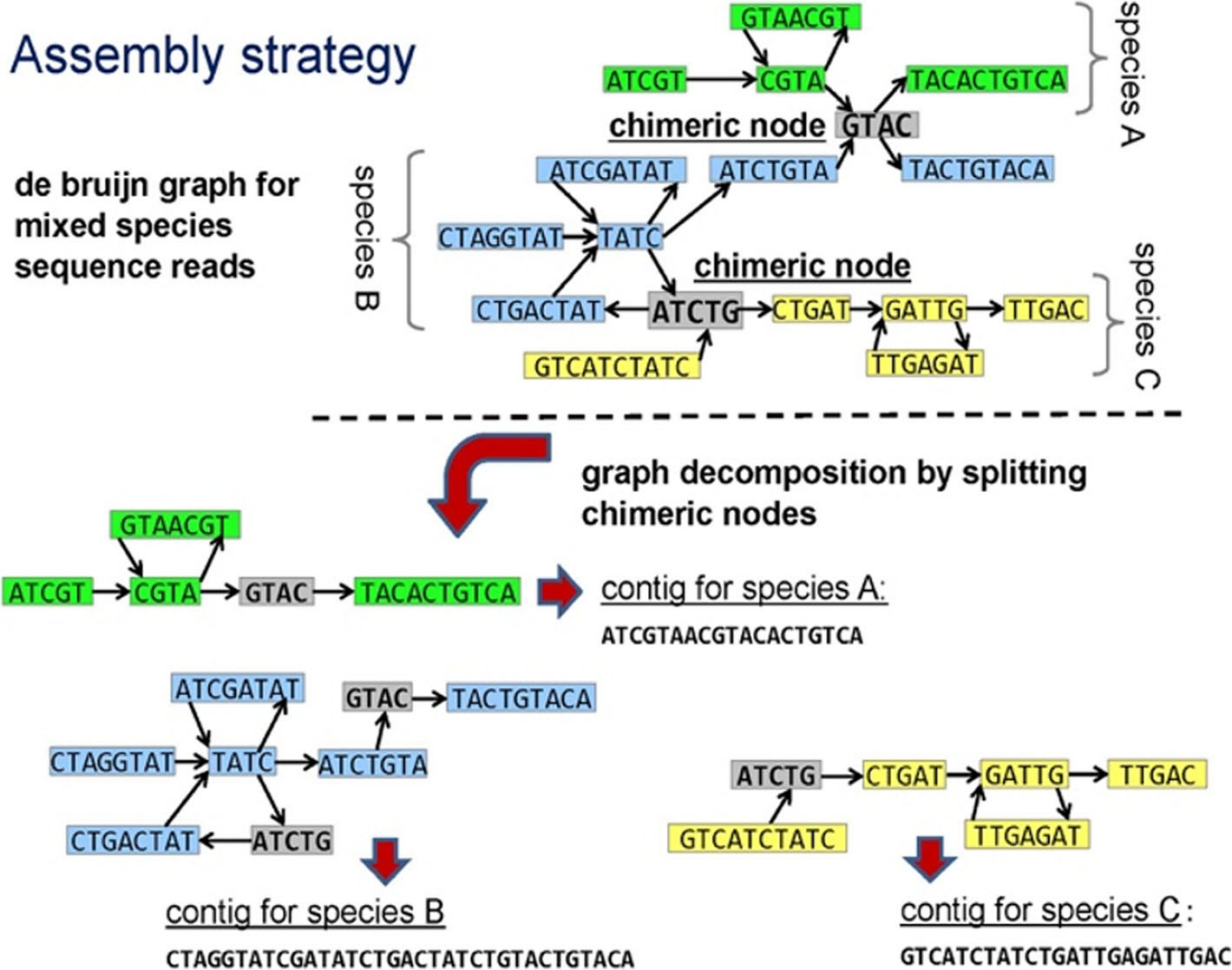
Assembly strategy for MetaVelvet

To address this challenge, MetaVelvet [2] was developed as an extension to the de Bruijn graph (dBG)-based de novo assembler Velvet [3], for the assembly of short-read metagenomic WGS data. MetaVelvet constructs a dBG for the mixed sequence reads of the multiple species, which is then partitioned into subgraphs for the single species. The multispecies dBG is partitioned at certain nodes, called chimeric nodes. A chimeric node corresponds to a stretch of nucleotide sequence in common between two evolutionarily similar species, and has two incoming and two outgoing nodes, representing the diverging sequences [2]. A graph containing such a chimeric node is partitioned into two subgraphs with one pair of incoming and outgoing nodes each (Fig. 2). To pair up the incoming and outgoing nodes, MetaVelvet uses node coverage differences.

State-of-the-art approaches to WGS metagenomic data assembly includes several dBG-based approaches, such as metaSPAdes [4] and Megahit [5]. MetaSPAdes is an extension to the original SPAdes [6] with modifications and new functions to support metagenome assembly. Megahit uses succinct dBGs [7] for assembly to handle the increasingly large and complex metagenomic datasets produced by the latest sequencing technologies. Based on Burrows-Wheeler Transform [8] succinct dBGs require less memory; e.g., 300 GB of human genome data can be stored in as little as 2.5 GB of memory. Megahit also implements a multiple k-mer strategy where an initial succinct dBG of order *k* is built from the reads, and the contigs are assembled based on the succinct dBG of order *k-d*, with *d* being the k-mer step size.

While this work focuses on de novo assemblers for short-read sequencing, recent advances in long-read sequencing technologies from companies such as Pacific Biosciences and Oxford Nanopore have enabled long-read sequencing of metagenomic samples [9, 10] and present a promising development in metagenomic analysis. However, short-read sequencing still has many advantages, including higher accuracy, higher throughput, lower cost, and well-established data analysis pipelines.

While MetaVelvet does not have the disadvantage of requiring reference genomes, the partitioning of chimeric nodes, i.e., how to determine which incoming and outgoing edge pairs represent sequences from the same species when there are two possible pairings, poses a key challenge (Fig. 2). As mentioned above, MetaVelvet considers node coverage, but not sequence information. Afiahayati et al. [11] developed MetaVelvet-SL, an extension to MetaVelvet using supervised learning, to improve the accuracy of node partitioning prediction. In MetaVelvet-SL, read coverage as well as sequence information (dinucleotide frequencies) on the incoming and outgoing nodes and the candidate chimeric node (Fig. 2) are used as features for a support vector machine (SVM) classifier that is trained to determine how to partition the dBG at the candidate chimeric node. While the consideration of dinucleotide frequencies does improve the chimeric node prediction accuracy, dinucleotide frequencies alone do not convey all sequence information that may help with chimeric node prediction as they do not cover patterns that exist over a range longer than two nucleotides.

Genome sequences contain species-specific patterns, and in particular, microbial DNA is known to show long-range correlations and patterns [12, 13]. For a machine learning algorithm to utilize long-range patterns that exist in microbial DNA to help partitioning a multi-species dBG into single-species subgraphs, transformation techniques for sequence data [14, 15] can be applied to the node sequences to engineer features that incorporate patterns at various scales. However, the kind of transformation and scale of sequence patterns that would be the most suitable to train a machine learning algorithm for the partitioning of a dBG remains unclear. To avoid the difficult task of determining suitable features for the machine learning model, we can allow machine learning algorithms to themselves discover appropriate features or representations. Deep learning, one of the latest developments in the field of machine learning, has been shown to outperform most traditional approaches [16], especially in tasks where spatial relationships exist in the data, such as images and text. One of the key benefits of deep learning models is that they can automatically extract suitable representations from raw data for a given problem, eliminating the need for handcrafting features based on expert knowledge in traditional supervised learning approaches.

In this study, we aimed to improve the performance of the metagenome assembler MetaVelvet-SL in predicting the partitioning of a dBG at chimeric nodes by using a deep learning-based model. The algorithm, which we termed MetaVelvet-DL, predicts the incoming–outgoing node pairs of a chimeric node that are from the same species based on raw nucleotide sequences of the chimeric and incoming and outgoing nodes, and their read coverage information. We designed a deep learning architecture that consists of embedding, convolutional, max-pooling, and bidirectional long short-term memory (LSTM) layers. LSTM is a type of recurrent neural network [17], where input data are fed sequentially to an LSTM unit. At each timestep, there are three important values to the LSTM unit: 1) input at the current timestep, 2) output of the LSTM at the previous timestep, and 3) the previous cell state. The cell state is responsible for remembering the spatial dependencies of neighboring inputs, the input and forget gates update the cell state, and the output gate controls the extent to which the output at each timestep is affected by the cell state. These mechanisms allow LSTM to remember longer-range interactions and avoid the vanishing and exploding gradient problems often encountered in traditional recurrent neural networks. LSTM has shown great promise in applications where data are sequential in nature, such as in speech recognition, protein homology detection, and protein subcellular localization [18–20]. As WGS metagenomics data generally cover complete genome sequences and spatial correlation and patterns can occur in concert with upstream or downstream sequences, we utilized bidirectional LSTM (biLSTM) [21] to incorporate up- and downstream sequence information in the output of a biLSTM unit. Finally, we compared assembly performance of MetaVelvet-DL with that of MetaVelvet-SL and the state-of-the-art assemblers Megahit and metaSPAdes, using datasets from Critical Assessment of Metagenome Interpretation (CAMI) [22]. The results showed that MetaVelvet-DL produces assemblies that have a lower rate of chimeric assembly and longer contigs.

## Results and discussion

We first present the accuracy of the deep learning classification model compared to the SVM model used in MetaVelvet-SL, followed by a comparison of the assembly results between MetaVelvet-DL, MetaVelvet-SL, MetaVelvet-DL-Kraken, and Megahit, and metaSPAdes, all with a k-mer size of 31 bp. MetaVelvet-DL-Kraken is a MetaVelvet-DL model trained on bacterial species predicted by the taxonomic identification software, Kraken [23]. A comparison of the assembly results of MetaVelvet-DL-Marmoset and SL-Marmoset are presented to show the robustness of the DL models, and finally, the results of MetaVelvet-DL and MetaVelvet-SL and Megahit on the CAMI medium-complexity dataset are also presented.

### Classification model performance

The prediction results of the trained MetaVelvet-SL and MetaVelvet-DL models for the validation dataset are provided in Table 1. MetaVelvet-DL showed higher sensitivities and specificities than MetaVelvet-SL for all three classes. When considering only true chimeric nodes and the rate at which they were incorrectly partitioned, i.e., true class 1 predicted as class 2 and true class 2 predicted as class 1, MetaVelvet-DL has a class 1 ➔ class 2 error rate of 14.8% and a class 2 ➔ class 1 error rate of 6.8%, whereas MetaVelvet-SL has the error rates 19.7 and 18.5%, respectively.

**Table 1:** Validation dataset accuracy for MetaVelvet-DL and MetaVelvet-SL

	MetaVelvet-DL	MetaVelvet-SL
Accuracy	78.3%	57.5%
Class 1 Sensitivity	75.5%	64.3%
Class 1 Specificity	90.0%	80.7%
Class 2 Sensitivity	85.4%	66.8%
Class 2 Specificity	86.2%	71.0%
Class 3 Sensitivity	73.9%	41.4%
Class 3 Specificity	91.3%	84.7%

### CAMI low-complexity dataset assembly

The MetaVelvet-DL and SL predictions were compared to the true labels (Table 2). The overall prediction accuracy on the CAMI low-complexity dataset was 55.6% for MetaVelvet-DL and 46.1% for MetaVelvet-SL. MetaVelvet-DL performed better than MetaVelvet-SL in all measures (sensitivity, specificity, and balanced accuracy) for each class, except for class 1 sensitivity, for which the two methods were comparable.

**Table 2:** MetaVelvet-DL and -SL prediction accuracies on the CAMI low-complexity dataset

	MetaVelvet-DL	MetaVelvet-SL
Accuracy	55.6%	46.1%
Class 1 Sensitivity	35.9%	26.2%
Class 1 Specificity	82.9%	87.7%
Class 1 Balanced Accuracy	59.4%	56.9%
Class 2 Sensitivity	64.5%	54.5%
Class 2 Specificity	75.5%	63.6%
Class 2 Balanced Accuracy	70.0%	59.1%
Class 3 Sensitivity	60.1%	51.2%
Class 3 Specificity	70.1%	63.1%
Class 3 Balanced Accuracy	65.3%	57.2%

We processed the CAMI low-complexity dataset assemblies generated by MetaVelvet-gold standard, MetaVelvet-DL, MetaVelvet-DL-Kraken, MetaVelvet-SL, Megahit, and metaSPAdes with MetaQUAST. We present the Total Contig Length, Number of Contigs (> 500 bp), N50 (contigs > 500 bp), Misassembled contig length ratio (Misassembled contigs length / Total length), and Genome fraction (%) calculated by MetaQUAST in Table 3. The full MetaQUAST report can be found in Supplementary A. We BLASTed the assemblies to the gold standard reference genomes and included in Table 3 to obtain the total chimeric contig length and chimeric contig length ratio, as well as the candidate chimeric node prediction accuracy (not for Megahit and metaSPAdes).

**Table 3:** CAMI low-complexity dataset assembly results for the assemblers evaluated

	MetaVelvet-gold standard	MetaVelvet-DL	MetaVelvet-DL-Kraken	MetaVelvet-SL	Megahit	metaSPAdes
N50 (> 500 bp)	5153	4130	4858	3830	5533	31,230
# Contigs (> 500 bp)	21,111	24,781	23,123	26,029	31,995	19,701
Total contig length	61,955,806	62,484,115	61,458,347	61,695,311	93,724,510	82,094,551
Misassembled contig length ratio	6.26e-3	8.52e-3	8.79e-3	8.66e-3	5.72e-3	7.36e-2
Genome fraction (%)	36.1	38.9	36.8	39.8	64.6	60.4
Total chimeric contig length	12,452,954 (20.1%)	12,750,152 (20.4%)	11,904,987 (19,4%)	12,417,820 (20.1%)	26,182,872 (27.9%)	42,291,210 (51.6%)
Chimeric class accuracy	26,315/26,315 (100%)	14,622/26,315 (55.6%)	15,493/26,315 (58.7%)	12,143/26,315 (46.1%)	NA	NA

Of all the assemblers, metaSPAdes had the highest N50 values from contigs of > 500 bp and the smallest number of contigs over 500 bp. However, metaSPAdes also has the highest misassembled contig length ratio (0.0736) that is an order higher than the other assemblers and the highest total chimeric contig length and ratio (0.516). Megahit had the largest number of contigs over 500 bp, total contig length, and genome fraction, but had the second highest total chimeric contig length and ratio (0.279). The MetaVelvet-based assemblers in general had much lower total contig lengths, and N50, and genome fraction than either Megahit or metaSPAdes but had much lower total chimeric contig lengths and ratios, with MetaVelvet-DL-Kraken having the lowest ratio at 0.194. They also had comparable misassembled contig length ratios to that of Megahit and much lower than that of metaSPAdes.

Within the MetaVelvet-based assemblers, MetaVelvet-gold standard had the largest N50 and the lowest misassembled contig length ratio as expected for a model using the gold standard labels. MetaVelvet-DL had the next lowest misassembled contig length ratio, followed by MetaVelvet-SL and then MetaVelvet-DL-Kraken. However, the trend is not as clear with total chimeric contig length, where MetaVelvet-DL-Kraken had the lowest ratio of total chimeric contig length and MetaVelvet-DL had the highest ratio, although all ratios for the MetaVelvet-based assemblers are very similar to each other. For chimeric class accuracy, the DL models also had much higher accuracy than that of the SL model. MetaVelvet-DL-Kraken, however, does have slightly higher prediction accuracy than that of MetaVelvet-DL.

**Fig. 3:**
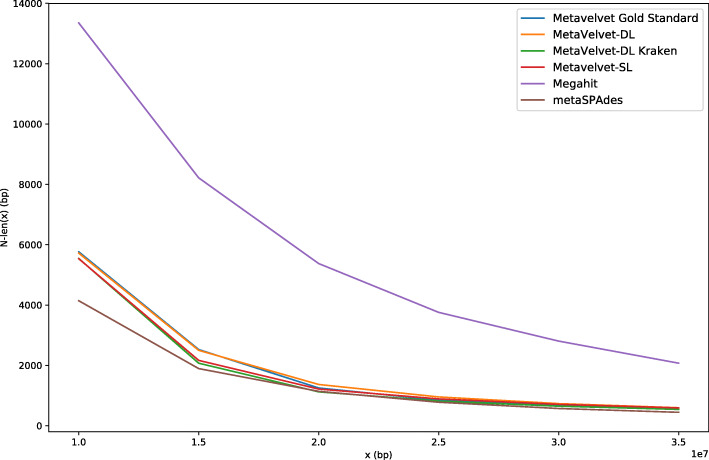
N-len(x) plots for MetaVelvet-DL, MetaVelvet-DL-Kraken, MetaVelvet-SL, MetaVelvet-gold standard, metaSPAdes, and Megahit

As discussed earlier, looking only at N50 value does not reflect the total length of contigs assembled. For a more unbiased comparison of assembly quality, we removed the chimeric contigs and plotted the N-len(*x*) scores for all six assemblies from *x* = 1e7 bp to *x* = 3.5e7 bp (Fig. 3).

When compared based on N-len(*x*) scores, Megahit produced significantly longer contigs than the MetaVelvet-based assemblers and metaSPAdes at all values of *x*, with metaSPAdes having the lowest N-len(*x*) scores at all *x* among all the assemblers. N-len(*x*) scores for MetaVelvet-DL were higher than those for MetaVelvet-SL at all values of *x*, and closely approximated those of MetaVelvet-gold standard, even having longer length in some regions of *x*. It should be noted that while MetaVelvet-DL-Kraken had the lowest N-len(*x*) values among all MetaVelvet assemblers, it still had higher N-len(*x*) values than metaSPAdes at all values of *x*, and this was achieved while having incomplete information on the bacterial species in the CAMI dataset based on predictions by Kraken.

The bacterial families in the CAMI low-complexity and the marmoset rectal datasets are shown in Table 4, and the MetaVelvet-DL-Marmoset and SL-Marmoset assembly results using the mismatched marmoset training data are shown in Table 5. We can see that the classification accuracies are lower than those models using either gold standard-generated or Kraken-generated species. However, the deep learning model still had much higher accuracy than that of the SVM model. As expected, MetaVelvet-DL-Marmoset had higher total chimeric contig length and misassembled contig length ratio than those of MetaVelvet-DL and MetaVelvet-DL-Kraken. When compared with metaSPAdes, MetaVelvet-DL-Marmoset still showed more accurate results in terms of the proportion of chimera contigs length and misassembled contig length ratio, showing the robustness of the deep learning model to variations in the training dataset.

**Table 4:** Family-level comparison of bacterial contents in the CAMI low-complexity and marmoset rectal datasets

CAMI	Marmoset
Anaeroplasmataceae	Actinomycetaceae
Chitinophagaceae	Bacteroidaceae
Clostridiaceae	Clostridiaceae
Chromobacteriaceae	Coriobacteriaceae
Comamonadaceae	Corynebacteriaceae
Desulfobacteraceae	Enterobacteriaceae
Flavobacteriaceae	Erysipelotrichaceae
Intrasporangiaceae	Lachnospiraceae
Oxalobacteraceae	Lachnospiraceae
Peptostreptococcaceae	Oscillospiraceae
Proteinivoraceae	Peptostreptococcaceae
Pseudomonadaceae	Porphyromonadaceae
Rhodobacteraceae	Prevotellaceae
Thermosporotrichaceae	Rikenellaceae
Veillonellaceae	Ruminococcaceae
Xanthomonadaceae	Streptococcaceae
	Veillonellaceae

**Table 5:** CAMI low-complexity dataset assembly results with MetaVelvet models trained with a dataset generated from mismatched reference genomes

	MetaVelvet-DL-Marmoset	MetaVelvet-SL-Marmoset
N50 (> 500 bp)	6732	6599
# Contigs (> 500 bp)	23,188	23,299
Total contig length	66,787,785	66,053,025
Misassembled contig length ratio	2.17e-2	1.11e-2
Genome fraction (%)	40.5	42.1
Total chimeric contig length	15,671,826 (23.5%)	17,648,716 (26.7%)
Chimeric class accuracy	11,543/26,315 (43.9%)	7784/26,315 (29.6%)

### CAMI medium-complexity dataset assembly

To test the proposed DL models on more complex datasets, we trained from the gold standard species list a DL and an SL model and assembled the CAMI medium complexity dataset. The MetaQUAST and BLAST statistics can be found in Table 6. The full MetaQUAST report can be found in Supplementary B. From Table 6, we can see that both MetaVelvet-based assemblers outperformed Megahit in terms of lower total chimeric contig lengths. However, Megahit has much higher N50, total contig lengths, misassembled contig length ratio, and genome fraction. Comparing the two MetaVelvet-based assemblers, the N50 values and total chimeric contig lengths were very comparable, with MetaVelvet-DL having slightly higher N50 values and longer assembled contigs. MetaVelvet-DL had slightly higher total chimeric contig length and ratio but have a much lower misassembled contig length ratio according to MetaQUAST. It should be noted that the reason for the poor comparison to Megahit is that despite using 200GB memory, the MetaVelvet-based assemblers could build their de Bruijn graphs with only part of the reads in the CAMI medium complexity dataset due heavy memory requirements. Furthermore, it should be noted that with the same amount of memory, metaSPAdes was unable to assemble the CAMI medium complexity dataset, failing to produce any output based on partial reads.

**Table 6:** CAMI medium-complexity dataset assembly result with MetaVelvet models trained with training dataset generated from Kraken-predicted species genomes

	MetaVelvet-DL	MetaVelvet-SL	Megahit
N50 (> 500 bp)	997	991	2682
# Contigs (> 500 bp)	47,931	47,917	106,769
Total contig length	77,284,835	77,094,128	242,383,239
Misassembled contig length ratio	8.69e-3	1.10e-2	2.39e-3
Genome fraction (%)	14.6	14.6	45.3
Total chimeric contig length	9,796,796 (12.7%)	9,606,278 (12.5%)	13,140,546 (18.0%)

**Fig. 4:**
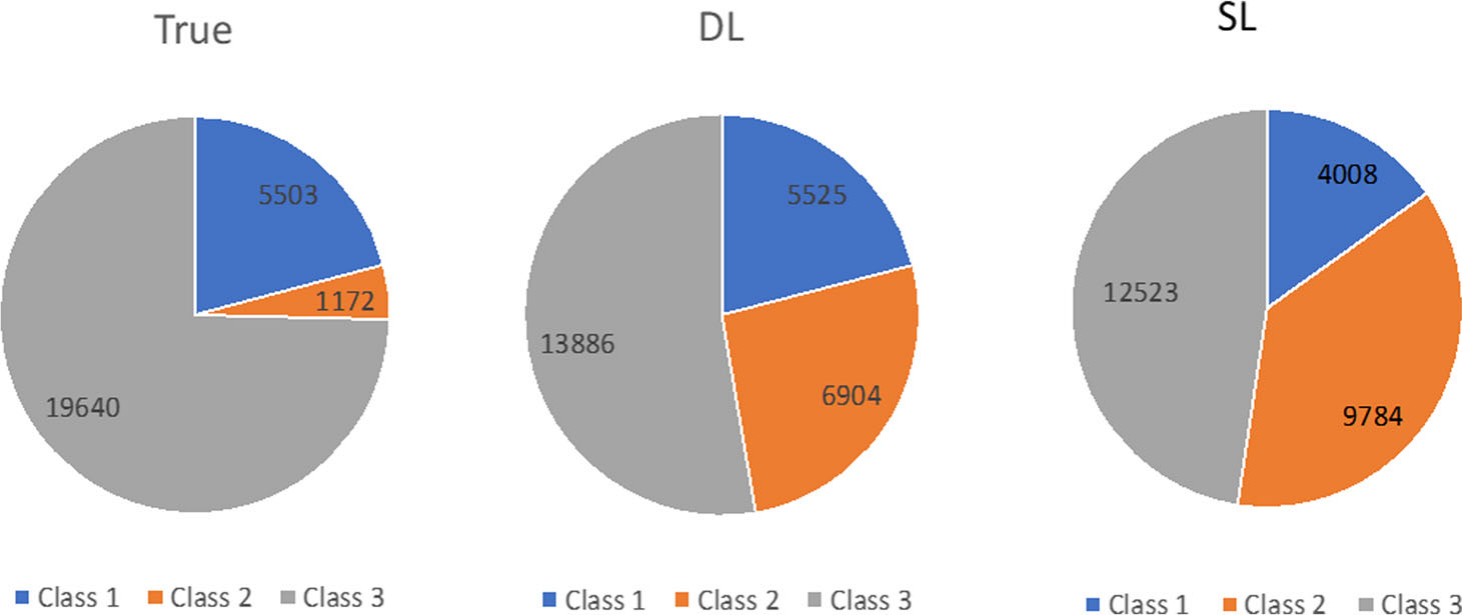
Distributions of true, DL, and SL-predicted labels of candidate chimeric nodes for the CAMI low-complexity dataset

In Fig. 4, we present a comparison of the distributions of true, DL, and SL-predicted class labels for the candidate chimeric nodes in dBG constructed from the CAMI low-complexity data. Based on the true labels, 3/4 of the candidate chimeric nodes were not chimeric, but repeat nodes. Class 1 true chimeric nodes were nearly 5 times more frequent than class 2 true chimeric nodes. As for MetaVelvet-DL, class 1 labels were the same amount as in the true labels, whereas class 2 labels were close to 6 times higher and class 3 labels nearly 30% less than those in the true labels.

It should be noted that the training datasets may contain some mislabeled samples. From Table 3, we can see that MetaVelvet-gold standard assembly, which used the true class labels for the partitioning of candidate chimeric nodes, also has chimeric assemblies. Besides rare cases of higher-connectivity nodes, partitioning the candidate chimeric nodes according to the true labels obtained by BLAST should have drastically reduced the chimeric rate. However, as can be seen from Table 3, the chimeric rate of MetaVelvet-gold standard is comparable to those of the other MetaVelvet assemblies. For MetaVelvet-DL, we used the same labeling scheme as used in MetaVelvet-SL. However, this scheme only looks at the highest scoring hit and does not consider any other BLAST hits, i.e., one or more of the HCI, HCO, LCI, LCO, and candidate chimeric nodes may be in fact be chimeric nodes. To show this, we created a dBG from a small set of reads simulated from the CAMI low-complexity reference genomes, with a total of 9247 samples of candidate chimeric nodes. The samples were labeled according to the MetaVelvet-SL labeling scheme and for each sample, we checked the chimeric status of the candidate chimeric, HCI, LCI, HCO, and LCO nodes. Out of the 9247 samples, 512 contained at least one node with a chimeric sequence, 401 of which are labeled as class 3. Since class 3 candidate chimeric nodes are not partitioned, this leads to chimeric contigs in the final assembly. While some of the chimeric node sequences in the samples are the results of the original Velvet assembly and cannot be eliminated without overhauling the entire Velvet implementation, we may be able to use the additional BLAST hits to reduce the chimeric sequences contributed to the final assembly for other samples. For example, using the original MetaVelvet-SL labeling scheme, if the highest-scoring hits for each of the four incoming and outgoing nodes of a sample would be mapped to the same species, this sample would be labeled as class 3. However, if the remaining hits for the HCI and HCO nodes contain a match to a different species, whereas all hits for the LCI and LCO nodes point to the same species, the candidate chimeric node can be relabeled as class 1, so that the subgraph containing the LCI and LCO nodes would be from the same species. In the training of the deep learning models, we used a balanced training set with equal numbers of all three classes. However, Fig. 4 shows that in an actual assembly for a metagenomic sample, the distribution can be dominated by class 3 nodes and class 2 nodes can be few. Furthermore, Fig. 4 also shows that MetaVelvet-DL does recognize the large proportion of class 3 nodes in the test dataset and has a class distribution closer to the true distribution than does MetaVelvet-SL.

**Fig. 5:**
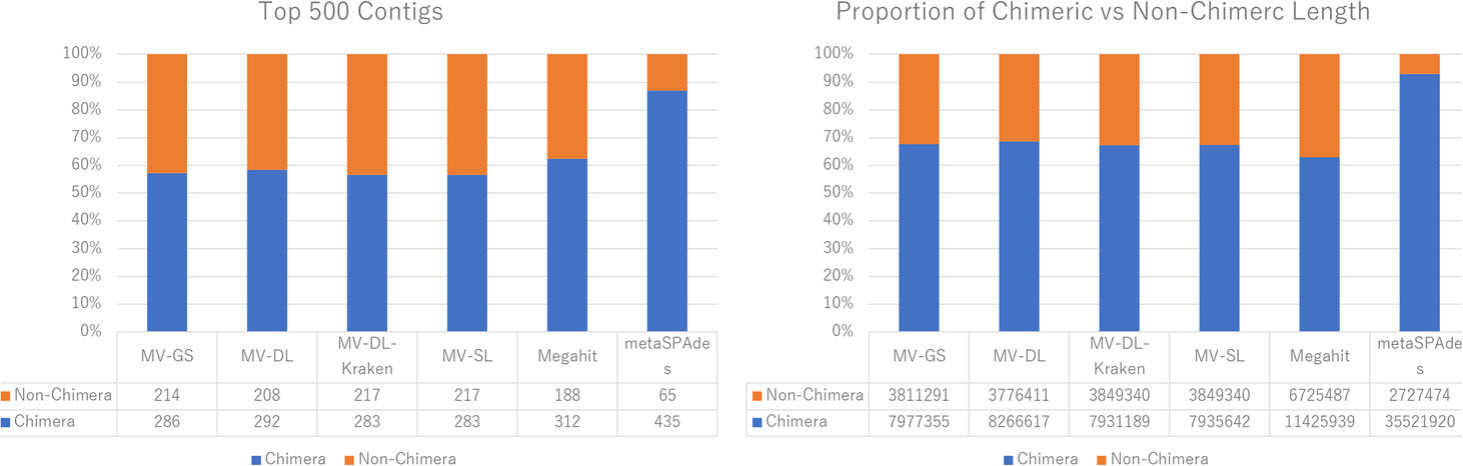
Proportion of top 500 contigs that are chimeric, and chimeric-to-total contig length ratio for the top 500 contigs for MetaVelvet-gold standard, MetaVelvet-DL, MetaVelvet-DL-Kraken, MetaVelvet-SL, Megahit, and metaSPAdes

In Fig. 5, we selected the top 500 contigs by length from each assembler and looked at the proportion of chimeric contigs in the top 500 contigs for the CAMI low-complexity dataset assemblies. The Velvet-based assemblers all had similar proportions of chimeric assembly at approximately 60%, as well as that of Megahit. MetaSPAdes had the highest proportion of chimeric contigs in the top 500 contigs at 87%. The difference between the Velvet-based assemblers and metaSPAdes becomes even more apparent when looking at the chimeric-to-total length ratio for the top 500 contigs, which are almost at 70% for the Velvet-based assemblers, but 93% for metaSPAdes. For CAMI medium-complexity dataset, the MetaVelvet-DL assembly produced 273 chimeric contigs in the top 500 contigs, with 5,547,178 bps out of the 10,081,697 bps (55.0%) total top 500 contig lengths being chimeric. For the MetaVelvet-SL assembly, 269 of the top 500 contigs were chimeric, with 5,452,132 bps of the 9,967,149 bps (54.7%) total top 500 contig lengths being chimeric.

It should be noted that the deep learning model can lead to overfitting, as evident from the decrease in accuracy between the validation set (Table 1) and the test set (Table 2). However, as shown in Table 3, even with overfitting, MetaVelvet-DL produced the best assembly in terms of low chimeric assembly. Furthermore, the DL model trained with unrelated marmoset metagenomic data also produced a better assembly than its SL counterparts and Megahit. This indicates that the DL model may have found some non-species-specific sequence features that could be generalized to other datasets with different bacterial species content.

While MetaVelvet-DL was shown to produce accurate assemblies and to be robust to mismatches between training and test metagenome data, this model still has some limitations, and we would like to further improve the assembler in future. Because of the increasing size of metagenomic data, memory usage in Velvet-based assemblers has become a bottleneck. The adoption of more memory-efficient indexing methods is urgently needed to improve the ease of use of MetaVelvet-DL on systems with fewer resources. While MetaVelvet-DL outperformed well despite the exclusion of higher-order connectivity nodes, the inclusion of such nodes is another approach for further improving the metagenomic assembly quality. One way to treat a node with higher connectivity is to decompose it into all possible incoming and outgoing pairs. The classifier can then be used to predict the label of each possible pair, and the resulting labels can be aggregated to make a final decision on how to partition the higher-connectivity node. Finally, we adopted the MetaVelvet-SL approach to label candidate chimeric nodes in the training data. The MetaVelvet-SL labeling scheme considers only the best BLAST hit and ignores any additional hits to other species, which may be a cause of chimeric contigs in the MetaVelvet-gold standard assemblies. We would like to explore other labeling schemes in future.

## Methods

In the following sections, we will first provide a brief review of MetaVelvet-SL to establish the framework MetaVelvet-DL is built upon and reformulate the dBG partitioning problem as a three-class classification problem where the classes represent possible ways of partitioning a dBG. The proposed deep learning architecture uses a one-dimensional convolutional neural network (1D CNN) and biLSTM networks to predict class labels of candidate chimeric nodes.

### Overview of MetaVelvet-SL

MetaVelvet-SL first constructs a dBG with Velvet, and performs simplification and error removal of tips, bubbles, and erroneous connections using Velvet functions [3]. However, typically, there remain nodes with multiple incoming and outgoing edges that could not be resolved to a single path. These chimeric nodes represent possible repeat regions that occur at multiple locations in a genome and are confirmed based on the actual read coverage and the expected coverage. However, in the assembly of multispecies genomic data, such chimeric nodes do not necessarily represent repeat regions, but may represent a stretch of nucleotide sequence that is evolutionarily conserved in different species.

**Fig. 6:**
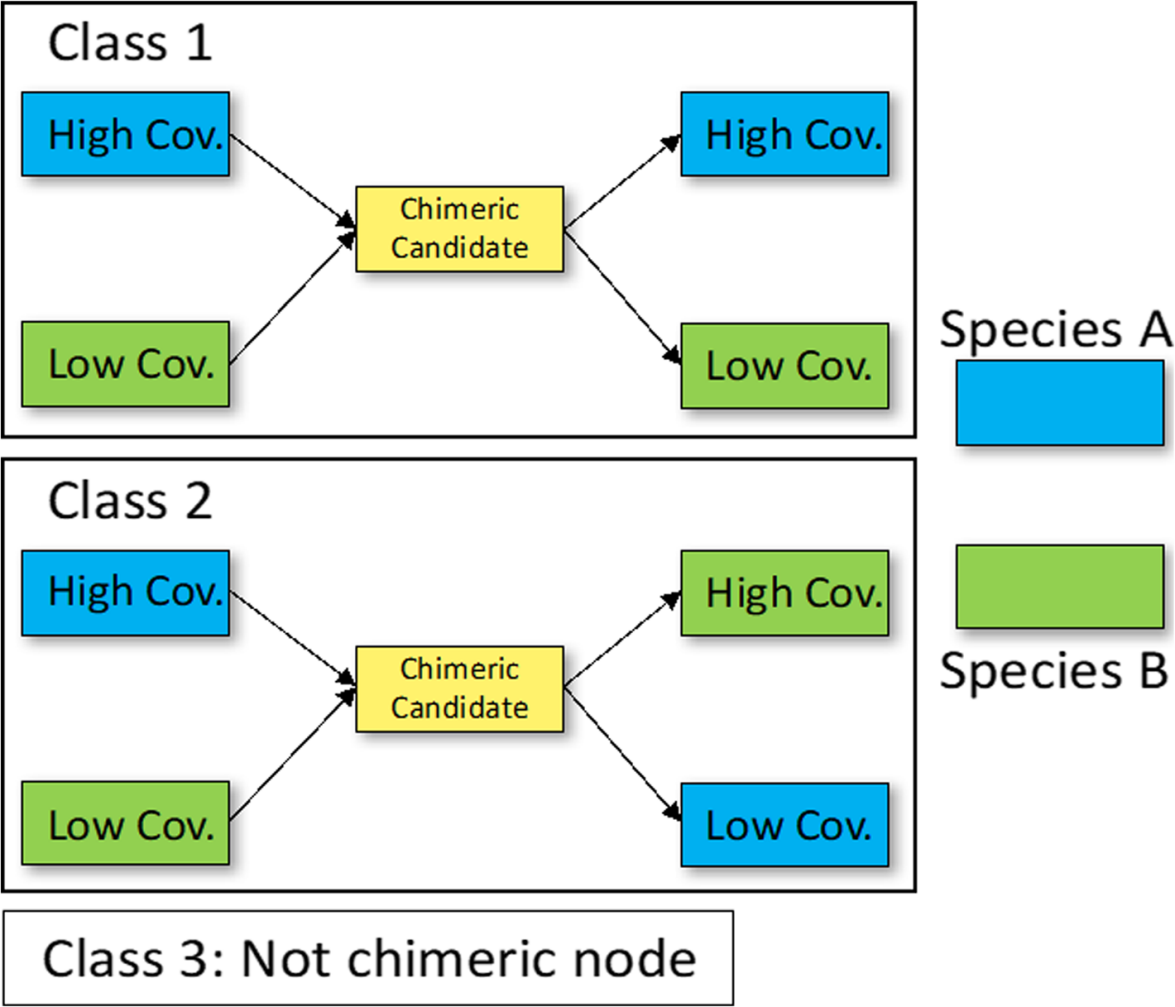
The three-class classification problem for graph partition in MetaVelvet-DL

In MetaVelvet and MetaVelvet-SL, the dBG constructed in Velvet is assumed to be composed of subgraphs that represent the individual species in a metagenomic sample. Therefore, one only needs to partition the multi-species dBG at the correct nodes to construct subgraphs and single-species contig assemblies. In MetaVelvet, partitioning is performed by identifying read coverage peaks, where each peak is assumed to represent one species in the microbial community. Each node is then assigned to a species, and a subgraph is formed by partitioning adjacent nodes having the same species assignment. Repeat nodes are distinguished from chimeric nodes by pair-end read mapping and coverage differences. In MetaVelvet-SL, a candidate chimeric node is defined as a node with two incoming and two outgoing nodes, which are labeled as higher- and lower-coverage incoming and outgoing nodes. There are three classes of possible arrangements at each candidate chimeric node as shown in Fig. 6: class 1, where the candidate node is chimeric, and the higher-coverage incoming and outgoing nodes belong to one species and the lower-coverage incoming and outgoing nodes belong to another species; class 2, where the candidate node is chimeric, and the higher-coverage incoming and lower-coverage outgoing nodes belong to one species and the lower-coverage and higher-coverage outgoings node belong to another; and class 3, where the candidate node is not chimeric, but a repeat node. MetaVelvet-SL uses a three-class SVM to predict the class labels of candidate chimeric nodes; in addition to the pair-end mapping and coverage information, which is also used in MetaVelvet, node sequence information in the form of dinucleotide frequencies is included in the SVM feature vector. MetaVelvet-SL performs metagenome assembly using the following steps:

1. Construct a dBG that consists of multi-species genomes using Velvet functions.
2. Generate a list of candidate chimeric nodes from the dBG constructed in step 1 and obtain the nucleotide sequences as well as pair-end mapping and coverage information for these nodes and their incoming and outgoing nodes.
3. Train a three-class classification SVM model and predict the class labels of candidate chimeric nodes using dinucleotide frequencies and pair-end mapping and coverage information as input features.
4. Partition the multi-species dBG into subgraphs at candidate chimeric nodes that have been classified as classes 1 and 2 in step 2.

The MetaVelvet-DL algorithm developed in this study followed the same steps as MetaVelvet-SL, but replacing the SVM model in step 3 with a deep learning architecture, taking advantage of the latter’s automatic feature extraction ability to consider long-range patterns in same-species sequences with the aim to resolve the graph partition problem. It should be noted that while candidate chimeric nodes with more than two incoming and outgoing nodes do exist, these account for only 1.79% of the candidate chimeric nodes in human gut microbiome [11]. Thus, in this study we focused on the modeling of nodes with two incoming and outgoing nodes. Another potential source of chimeric nodes is horizontal gene transfer between different species. While this phenomenon is not considered in this work, it is a future direction for the extension of this algorithm.

### Deep learning classification model

**Fig. 7:**
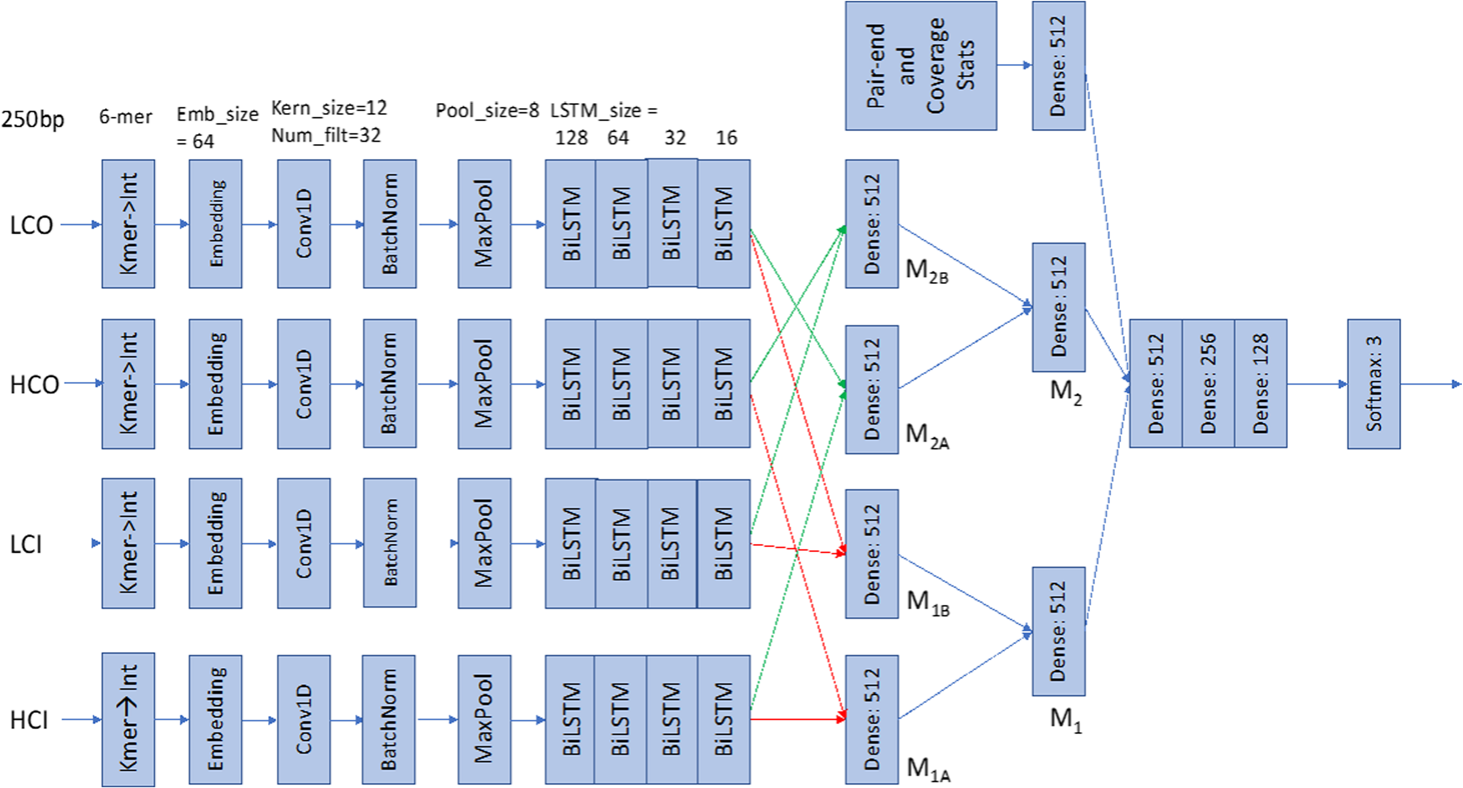
Deep learning architecture for candidate chimeric node classification in MetaVelvet-DL

We designed a deep learning architecture, illustrated in Fig. 7, to predict whether a candidate chimeric node is a true chimeric or a repeat node. The architecture follows directly from the structure of the problem itself: there are two incoming and two outgoing nodes, and we need to predict the incoming–outgoing nodes that form a correct pair, or whether none of the nodes do. To this end, we used one 1D convolutional layer and four biLSTM layers on each node sequence, except for the candidate node. The structure then combined the outputs of the biLSTM layers to represent both possible pairings and uses a series of fully connected layers to determine the correct partition method. Hereafter, the higher-coverage incoming node, lower-coverage incoming node, higher-coverage outgoing node, and lower-coverage outgoing node are denoted as HCI, LCI, HCO, and LCO nodes, respectively.

#### Input

The input to the deep learning architecture includes the sequences for a candidate chimeric node, the two incoming nodes, and the two outgoing nodes, and coverage information that includes:

1. Number of reads connecting the HCI and HCO nodes.
2. Number of reads connecting the HCI and LCO nodes.
3. Number of reads connecting the LCI and HCO nodes.
4. Number of reads connecting the LCI and LCO nodes.
5. Coverage ratios of the incoming nodes to the candidate chimeric node.
6. Coverage ratios of the outgoing nodes to the candidate chimeric node.
7. Coverage of the candidate chimeric node.
8. Length of the candidate chimeric node.

The coverage features are included as additional evidence for graph partitions. A higher number of pair-end reads mapped to a certain pair of incoming–outgoing nodes gives stronger evidence for partitioning. In cases where the incoming or outgoing nodes have highly similar sequences, coverage features can be weighted higher in the decision process. For each of the eight coverage features, a histogram is computed and divided into 10 equally spaced quantiles. The pair-end and coverage feature values of each candidate chimeric node are then one-hot-encoded according to their respective quantile and concatenated to form a binary vector. For sequence data, instead of assigning the four nucleotides ‘A’, ‘G’, ‘C’, and ‘T’ the numbers 1, 2, 3, and 4, or using one-hot encoding, we assigned all possible hexanucleotides a unique integer between 1 and 4096, e.g., ‘AAAAAA’ = 1, ‘AAAAAG’ = 2, and ‘TTTTTT’ = 4096. Accordingly, a 6-bp sliding window is slid from the 5′ to the 3′ end of a sequence at 1-bp step size, at each step assigning the observed hexanucleotide its corresponding integer to obtain a numeric representation of the nucleotide sequence. Using this representation allows us to include short-range patterns in the input data, while leaving sufficient freedom for the deep learning model to find an optimal representation for the classification problem. As input to the deep learning architecture, we extracted 250 bp from the 3′ end of the incoming nodes and 250 bp from the 5′ end of the outgoing nodes and converted the nucleotide sequences to integer sequences as described above. In case the length of a node was less than 250 bp, the integer sequences were padded with zeros so that all sequences have the same length.

#### Embedding layer

The first layer of the deep learning architecture proposed here is an embedding layer. The embedding layer is applied only to the integer-transformed nucleotide sequences of the candidate chimeric and incoming and outgoing nodes. In natural language processing, word embedding is a dense representation of words in the form of a numerical vector [24] and serves as a method for dimension reduction from the original word space and to encapsulate co-occurrence information [25]. In our architecture, we chose to implement an embedding layer that embeds each integer into a numerical vector of length 64, and the embeddings are learned during the training process.

#### Convolutional layers

After embedding, the dense representation of each of the incoming and outgoing nodes of a candidate chimeric node is passed through a 1D CNN layer. A nucleotide sequence is 1D data where spatial relationships exist on various scales. The 1D CNN is used to learn features present within the nucleotide sequences, taking advantage of its strength in identifying spatial patterns that exist within input data [26]. The 1D CNN filter is a sliding window that moves across the input data with a step size of 1, convolving the values of the window with the input within the window at each step and producing a 1D feature map as output. In the architecture proposed here, each of the four 1D CNN layers has 32 filters of size 64 × 12, where 64 is the size of the embedding layer output and 12 is the window length.

#### Batch normalization

Each 1D CNN layer is subjected to batch normalization, which transforms each activation in the output feature map from the 1D CNN layers such that they have zero mean and unit variance. This step can speed up the learning by using large learning rates [27].

#### Maxpooling

Maxpooling is applied to the batch-normalized feature maps. Maxpooling with 1D input is simply a moving window that takes the maximum value within the window at each step. Such an operation downsizes the input and reduces dimensionality, but still allows the most prominent features in each window to be observed [28]. In the architecture proposed here, we set the maxpooling size to 4.

#### BiLSTM

For each of the incoming and outgoing nodes, the output from the maxpooling layer is used as input to four consecutive biLSTM layers. As WGS metagenomics data generally cover complete genome sequences and spatial correlations or patterns can occur in concert with upstream or downstream sequences, we utilized biLSTM [21] to process each maxpooling output both forward and backward to incorporate up- and downstream sequence information in the output of a biLSTM unit. We stacked four biLSTM layers, where the input to the first layer is the output of the maxpooling layer, and the input of the subsequent layers is the output of the respective previous layers. Each subsequent layer will learn sequence patterns at a larger scale than the previous one. In the architecture proposed here, we used biLSTM layers of sizes 128, 64, 32, and 16.

#### Fully connected layers

Fully connected layers where all neurons in one layer are connected to all neurons in the next are used to aggregate all previous layers and to generate a nonlinear combination of the features learned in the previous layers. Let us first denote the network stacks described above from the integer sequence inputs to the embedding layer to the output of the final biLSTM layer for the HCI, LCI, HCO, and LCO nodes as M_HCI_, M_LCI_, M_HCO_, and M_LCO_, respectively. We then concatenate pairs of network stacks to obtain the following:

1. M_1A_: the concatenation of M_HCI_ and M_HCO_
2. M_1B_: the concatenation of M_LCI_ and M_LCO_
3. M_2A_: the concatenation of M_HCI_ and M_LCO_
4. M_2B_: the concatenation of M_LCI_ and M_HCO_

and the output of each concatenation is connected to a separate single layer of 512 neurons. The outputs of the single layers are further concatenated as follows:

1. M_1_: the concatenation of M_1A_ and M_1B_
2. M_2_: the concatenation of M_2A_ and M_2B_

where M_1_ and M_2_ correspond to class 1 and 2 pairings, respectively. The outputs of M_1_ and M_2_ are again each connected to a single layer of 512 neurons. The one-hot-encoded features of pair-end and coverage information are next used as inputs to a single layer of 512 neurons. The three layers are then concatenated and used as input to three fully connected layers of sizes 512, 256, and 128. The output of the final fully connected layer is then fed into a softmax layer with three outputs corresponding to the three classes.

The deep learning architecture presented above reflects two design goals. First, in dBGs constructed from metagenomic samples, we observed that node sequence lengths can range from tens to thousands of base pairs. To accurately capture sequence patterns at various resolutions, we included four biLSTM layers, each layer capturing increasingly longer-range patterns. Second, the architecture contains two large subnetworks that are concatenated in the fully connected layers, where each subnetwork represents one possible partition of a chimeric node. The fully connected layers then combine the subnetworks and read coverage information to decide on which is the most probable partition for a candidate chimeric node.

### Data

To compare the performance of the MetaVelvet-DL, MetaVelvet-SL, Megahit, and metaSPAdes assemblies, we used low- and medium complexity datasets from the first Critical Assessment of Metagenome Interpretation (CAMI) challenge, which is an effort to provide a standardized benchmark for comparing metagenomic data analysis tools. The low-complexity dataset comprises approximately 50,000,000 pair-end reads with Illumina HighSeq error profile from 40 microbial and viral genomes and 20 circular elements simulating a single sample, for a total of 15 Gb. The medium-complexity dataset covers 132 genomes and 100 circular elements simulating two samples, for a total of 40 Gb. In both datasets, the pair-end reads have read length of 150 bp, a mean insertion length of 270 bp, and a standard deviation of 27 bp.

### Training strategy for unknown bacterial species in a metagenomic sample

We generated a training set of candidate chimeric nodes, using gold-standard bacterial species genomes for the CAMI low-complexity dataset. A dBG was constructed at a k-mer size of 31 bp using 150-bp pair-end reads with a 270-bp insertion length and 50× coverage. The processing pipeline we used is as follows:

1. Use the gold standard list of bacterial species for CAMI low-complexity dataset.
2. Generate simulated pair-end reads from the reference genomes of the gold standard species using DWGSIM (https://github.com/nh13/DWGSIM).
3. Construct a dBG from the simulated data and identify the candidate chimeric nodes.
4. BLAST the candidate chimeric, incoming, and outgoing node sequences to the reference genomes to determine the partition class label for each candidate chimeric node.
5. Train the deep learning model with the simulated dataset.
6. Construct a dBG for the unknown metagenomic sample and identify the candidate chimeric nodes.
7. Predict the partition classes of the candidate chimeric nodes in the unknown metagenomic sample and partition the dBG accordingly.

From the above pipeline we created a training set of candidate chimeric nodes from dBGs that were constructed at a k-mer size of 31 bp, using 50 × −coverage, 150-bp pair-end reads with an insertion size of 270 bp simulated from the gold standard species list for CAMI low-complexity dataset. Based on the strategy used in MetaVelvet-SL, we BLASTed the node sequences to the reference genomes of the gold standard bacterial species to obtain the true labels of the candidate chimeric nodes for the training set. In MetaVelvet-SL, we considered the highest-ranking match for each of the incoming/outgoing node sequences. When the HCI and HCO nodes had the same highest-ranking match and the LCI and LCO had the same highest-ranking match different from that of HCI and HCO, we labeled the candidate chimeric node as class 1. When the HCI and LCO had the same highest-ranking match and the LCI and HCO had the same highest-ranking match different from that of HCI and LCO, we labeled the candidate chimeric node as class 2. All other candidate nodes were labeled as class 3. For the training set, 50,000 candidate chimeric nodes from each of classes 1, 2, and 3 were selected to create a training set of 150,000 samples. Another 1000 candidate chimeric nodes from each class were selected to create a validation set of 3000 samples, with a batch size of 100 samples over 256 epochs. The generated datasets are used to train and validate a deep learning model and an SVM model, and their resulting assemblies are denoted as MetaVelvet-DL and MetaVelvet-SL in the discussion. For comparison, we also performed an assembly of CAMI low-complexity dataset where we did not use either the SL or DL model predictions, but the true labels for the candidate chimeric nodes to partition the dBG. We refer to this as the MetaVelvet-gold standard in the discussion.

In real-world applications, a workflow to train a model for chimeric node prediction would first use a phylogenetic analysis software to predict the bacterial species in the unknown sample. Then, a training dataset would be created using simulations with the reference genomes of the predicted species. Here we trained a deep learning chimeric node prediction model using simulated read data generated from the genomes of a set of bacterial species predicted to be present in the CAMI low-complexity dataset by Kraken [28], which we will refer to as MetaVelvet-DL-Kraken. The training set contains 20,000 samples of each class, and the validation set contains 1000 samples of each class. The models were trained with a batch size of 100 samples over 100 epochs.

To demonstrate the robustness of MetaVelvet-DL, we also trained a deep learning and an SVM model using a highly mismatched training dataset generated from an unpublished microbial community found in a marmoset (*Callithrix jacchus*) rectal sample. The bacterial species families in the CAMI and marmoset datasets are shown in Table 4. It can be seen from the table that the two lists are mismatched, with very few common families in the two datasets. The training set has 20,000 samples of each of classes 1, 2, and 3, and 1000 samples of each class for validation. The models were trained with a batch size of 100 samples over 100 epochs. Hereafter, these models are referred to as MetaVelvet-DL-Marmoset and MetaVelvet-SL-Marmoset.

Finally, to evaluate the performance of MetaVelvet-DL on more complex datasets, we took one of the CAMI medium-complexity samples and trained DL and SL models with the same steps used to generate the MetaVelvet-DL model.

### Assessment

To bring focus to the quality of metagenome assemblies with respect to chimeric assembly, we used MetaQUAST [29] to provide a quantitative evaluation of various MetaVelvet-based assemblers with models trained with different training datasets, together with metaSPAdes and Megahit. In addition to providing standard quality statistics such as N50 and mapped genome fraction, MetaQUAST also includes metrics such as number of interspecies translocations and number of misassembled contigs [29], which are important in metagenome assembly.

We also BLASTed the assembled contigs to the gold standard reference genomes. If non-overlapping parts of a contigs are found to have hits in different species, then that contig is considered as a chimeric contig. By identifying the chimeric contigs through BLAST, we compared total chimeric contig lengths and proportions of chimeric contig length of each of the assemblers as a metric for assembly quality.

Another common metric for comparing genome assemblies is the N50 score, which is the length of the shortest contig where if all contigs longer than N50 summed together would account for 50% of the total assembly. However, comparing N50 of different assemblies is biased because of the differing total contig lengths in different assemblies. Therefore, we used the following generalized score, termed the N-len(*x*) score:

1 $$ \mathrm{N}\hbox{-} \mathrm{len}(x)=\left|{S}_i\right|\ni {\sum}_{j=1}^i\left|{S}_j\right|\ge x\;\mathrm{and}\;{\sum}_{j=1}^{i-1}\left|{S}_j\right|\le x, $$

where L is the total length of all contigs, *S*_*j*_ denotes the j-th contig in the total set of contigs sorted by length in a decreasing order, and | *S*_*j*_ | denotes its length. Based on this formulation, the N50 measure is simply a special case of the N-len(*x*) score where *x* = L/2 [2]. Using the N-len(*x*) score, we can compare the length of the shortest contig in the smallest set of contigs whose total length just exceeds the same value among all assemblers.

## Conclusion

We developed a dBG-based short-read de novo assembler that is an improvement over existing algorithms by introducing a deep learning model for a more accurate partition of multi-species dBGs into single-species subgraphs. The assembler, called MetaVelvet-DL, was shown to produce a lower ratio of misassembled contig length than those of MetaVelvet-SL and metaSPAdes, one of the state-of-the-art metagenome assemblers. MetaVelvet-DL assemblies also had higher N-len(*x*) scores than those of MetaVelvet-SL and metaSPAdes assemblies across a large range of assembly lengths, and closely approximated those of the gold-standard MetaVelvet assembly. While the proposed algorithm does not outperform the state-of-the-art algorithms in all aspects, we feel that the novel use of deep learning methods to learn representations directly from sequence data for dBG partitioning holds promise for future improvements.

## Availability and requirements

Project name: MetaVelvet-DL

Project home page: http://www.dna.bio.keio.ac.jp/metavelvet-dl/

Operating system: Platform independent

Programming language: Python 3

Other requirements: Tensorflow > = 1.0, Keras > = 2.0.5

License: GNU General Public License v2.0

Contact: yasu@bio.keio.ac.jp

Availability: The Python source code of MetaVelvet-DL is available at http://www.dna.bio.keio.ac.jp/metavelvet-dl/.

## Supplementary information

**Additional file 1.**

**Additional file 2.**

## Data Availability

The source code of MetaVelvet-DL is available at http://www.dna.bio.keio.ac.jp/metavelvet-dl/. CAMI datasets can be downloaded from https://data.cami-challenge.org/. The marmoset metagenomic data are currently not publicly available while the manuscript is still being prepared but can be made available upon request.

## Abbreviations

CAMI: Critical Assessment of Metagenome Interpretation
CNN: Convolutional neural network
LSTM: Long short-term memory
dBG: De Bruijn graph
SVM: Support vector machine
WGS: Whole genome sequencing

## References

[1] Ranjan R, Rani A, Metwally A, McGee HS, Perkins DL (2016). Advantages of whole genome shotgun versus 16S amplicon sequencing. Biochem Biophys Res Comun.

[2] Namiki T, Hachiya T, Tanaka H, Sakakibara Y (2012). MetaVelvet: an extension of velvet assembler to de novo metagenome assembly from short read sequence reads. Nucl Acids Res.

[3] Zerbino D, Birney E (2008). Velvet: algorithms for de novo short read assembly using de Bruijn graphs. Genome Res.

[4] Nerk S, Meleshko D, Korobeynikov A, Pevzner PA (2017). metaSPAdes: a new versatile metagenomic assembler. Genome Res.

[5] Li DH, Liu CM, Luo RB, Sadakane K, Lam TW (2015). MEGAHIT: an ultra-fast single-node solution for large and complex metagenomics assembly via succinct de Bruijn graph. Bioinformatics.

[6] Bankevich A, Nurk S, Antipov D, Gurevich AA, Dvorkin M, Kulikov AS, Lesin VM, Nikolenko SI, Pham S, Prjibelski AD (2012). SPAdes: a new genome assembly algorithm and its applications to single-cell sequencing. J Comput Biol.

[7] Bowe A, Raphael B, Tang J (2012). Succinct de Bruijn Graphs. Algorithms in bioinformatics.

[8] Burrow M, Wheeler DJ. A block-sorting lossless data compression algorithm. Digit SRC Res Rep. 1994; 1–18. Technical Report 124.

[9] Frank JA, Pan Y (2016). Improved metagenome assemblies and taxonomic binning using long-read circular consensus sequence data. Sci Rep.

[10] Brown BL, Watson M, Minot SS, Rivera MC, Franklin RB (2017). MinION nanopore sequencing of environmental metagenomes: a synthetic approach. Gigascience.

[11] Afiahayati S (2015). K, and Sakakibara Y. MetaVelvet-SL: an extension of the velvet assembler to a de novo metagenomics assembler utilizing supervised learning. DNA Res.

[12] Allen TE, Price ND, Joyce AR, Palsson B (2006). Long-range periodic patterns in microbial genomes indicate significant multi-scale chromosomal organization. PLoS Comput Biol.

[13] Li WT, Marr TG, Kaneko K (1994). Understanding long-range correlations in DNA-sequences. Phsica D.

[14] Arneodo A, Bacry E, Graves PV, Muzy JF (1995). Characterizing long-range correlations in DNA sequences from wavelet analysis. Phys Rev Lett.

[15] Sussilo D, Kundaje A, Anastassiou D. Spectrogram analysis of genomes. EURASIP J Adv Signal Process. 2004:790248 10.1155/S1110865704310048.

[16] Bengio Y, Courville A, Vincent P (2013). Representation learning: a review and new perspectives. IEEE Trans Pattern Anal Mach Intell.

[17] Hochreiter S, Schmidhuber J (1997). Long short-term memory. Neural Comput.

[18] Graves A, Mohamed AR, and Hinton G. Speech recognition with deep recurrent neural networks. Proc. IEEE Int. Conf. Acoust. Speech Signal Process., 2013, 6645–6649.

[19] Hochreiter S, Heusel M, Obermayer K (2007). Fast model-based protein homology detection without alignment. Bioinformatics.

[20] Thireou T, Reczko M (2007). Bidirectional long short-term memory networks for predicting the subcellular localization of eukaryotic proteins. IEEE/ACM Transac Comput Biol Bioinf.

[21] Graves A, Schmidhuber J (2005). Framewise phoneme classification with bidirectional LSTM and other neural network architectures. Neural Netw.

[22] Sczyrba A (2017). Critical assessment of Metagnome interpretation - a benchmark of metagenomics software. Nat Methods.

[23] Wood DE, Salzberg SL (2014). Kraken: ultrafast metagenomic sequence classification using exact alignments. Genome Biol.

[24] Mikolov T, Sutskever I, Chen K, Corrado G, Dean J (2013). Distributed representations of words and phrases and their compositionality. In Proc Adv Neural Inf Process Syst.

[25] Levy O, Goldberg Y (2014). Neural word embedding as implicit matrix factorization. In Proc Adv Neural Inf Process Syst.

[26] Lecun Y, Bottou L, Bengio Y, Haffner P (1998). Gradient-based learning applied to document recognition. Proc IEEE.

[27] Ioffe S, Szegedy C (2015). Batch normalization: accelerating deep network training by reducing internal covariate shift. Proc Int Conf Machine Learning.

[28] Scherer D, Muller A, and Behnke S. Evaluation of pooling operations in convolutional architectures for object recognition. In Proc. of the Intl. Conf. on Artificial Neural Networks, 2010; 92–101.

[29] Mikheenko A, Saveliev V, Gurevich A (2016). MetaQUAST: evaluation of metagenome assemblies. Bioinformatics.

